# Nrf2 activators for the treatment of rare iron overload diseases: From bench to bedside

**DOI:** 10.1016/j.redox.2025.103551

**Published:** 2025-02-14

**Authors:** Yimin Dong, Meng Zheng, Weizhong Ding, Hanfeng Guan, Jun Xiao, Feng Li

**Affiliations:** a Department of Orthopaedic Surgery, Tongji Hospital, Tongji Medical College, Huazhong University of Science and Technology, Wuhan, China; b Department of Orthopedics, Sun Yat-sen Memorial Hospital, Sun Yat-sen University, Guangzhou, China

**Keywords:** Nrf2 activators, Iron overload, Oxidative stress, Rare diseases, Iron metabolism

## Abstract

Iron overload and related oxidative damage are seen in many rare diseases, due to mutation of iron homeostasis-related genes. As a core regulator on cellular antioxidant reaction, Nrf2 can also decrease systemic and cellular iron levels by regulating iron-related genes and pathways, making Nrf2 activators very good candidates for the treatment of iron overload disorders. Successful examples include the clinical use of omaveloxolone for Friedreich's Ataxia and dimethyl fumarate for relapsing-remitting multiple sclerosis. Despite these uses, the therapeutic potentials of Nrf2 activators for iron overload disorders may be overlooked in clinical practice. Therefore, this study talks about the potential use, possible mechanisms, and precautions of Nrf2 activators in treating rare iron overload diseases. In addition, a combination therapy with Nrf2 activators and iron chelators is proposed for clinical reference, aiming to facilitate the clinical use of Nrf2 activators for more iron overload disorders.

## 1 Introduction

Many rare diseases are caused by mutations of genes involved in iron homeostasis regulation, leading to systemic iron overload, iron misdistribution or local deposition [1]. These diseases are incurable due to the lack of effective treatments. However, the recent approval of omaveloxolone for the treatment of Friedreich's Ataxia confirmed the efficacy of Nrf2 activators in iron-related rare disease [2]. Besides, dimethyl fumarate, another Nrf2 activator, has been used to treat multiple sclerosis [3], an iron-related rare neurological disease. In addition, clinical trials have shown good efficacy of bitopertin in treating Erythropoietic protoporphyria (EPP) [4,5], and bitopertin is also a potent Nrf2 activator [6]. These advances suggest that Nrf2 activators may be good candidates for the treatment of iron related-rare diseases.

In addition to its antioxidant effects, Nrf2 can reduce both cellular and systemic iron levels by regulating iron-related genes and pathways [7]. However, the iron-decreasing effects have received less attention in clinical practice. Since excess iron triggers the production of ROS that leads to oxidative damage, the antioxidant and iron-decreasing effects of Nrf2 provide tremendous clinical value in the treatment of iron overload disorders, especially in rare iron overload diseases. In this review, we discussed the potential use, possible mechanisms, and precautions of Nrf2 activators in treating these rare diseases. Besides, for severe iron overload disorders, we proposed a novel combination therapy involving iron chelator and Nrf2 activator for clinical reference. It is expected that more and more patients with rare iron overload diseases will benefit from the use of Nrf2 activators.

## 2 Systemic and cellular iron regulation

**Fig. 1:**
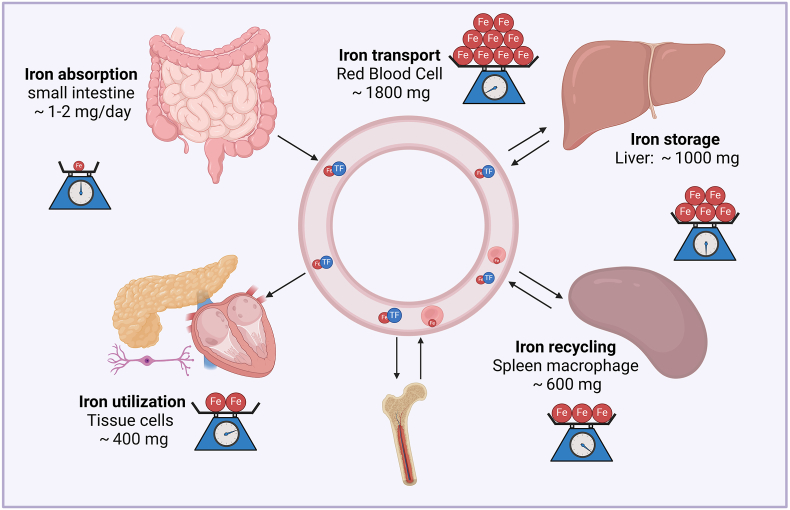
**Systemic and cellular maintenance of iron homeostasis.** Dietary iron is absorbed by the small intestine at doses of 1–2 mg daily to make up for non-specific iron loss from intestinal cell shedding or bleeding. For the systemic iron pool, a great proportion of iron (∼1800 mg) is used to constitute hemoglobin in red blood cells. When red blood cells become senescent, they are engulfed by macrophages and the iron (∼600 mg) is dynamically recycled, exported and transported to erythroid cells for a novel cycle of hemoglobinization. About 1000 mg of iron is reversibly stored in the liver and can be secreted at conditions of decreased serum iron. In mammals, iron cannot be secreted outside the body via controlled manners.

**Fig. 2:**
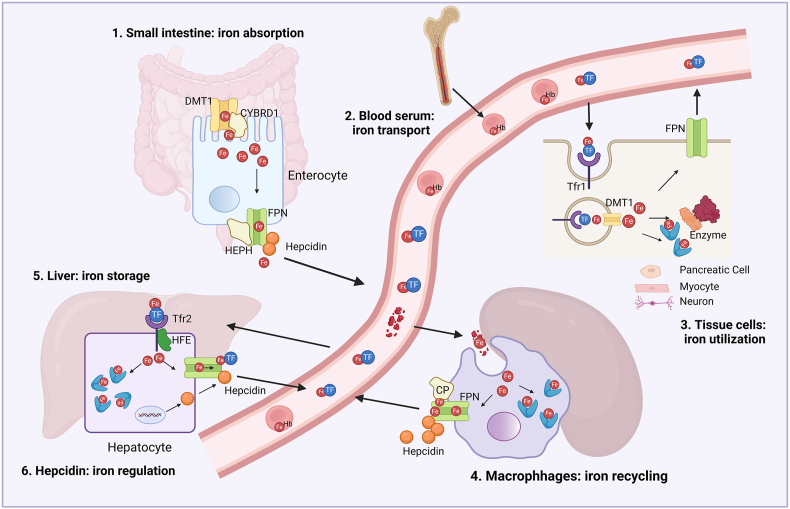
**Molecular regulation of iron homeostasis. 1. Iron absorption in the small intestine**. In the apical side of enterocytes, exogenous ferric iron is reduced by CYBRD1 to ferrous iron, and then transported into enterocytes by DMT1. At the basal side, iron is exported by FPN and oxidized by Hephaestin before entering the bloodstream; **2. Iron transport in the circulation.** In the serum, iron binds to transferrin and is delivered to various cells; In the bone marrow, iron is used to constitute hemoglobin during erythropoiesis and help to transport oxygen in mature red blood cells throughout the body; **3. Iron utilization in various tissue cells.** Tissue cells uptake iron via endocytosis after transferrin binding to its receptor Trf1. Intracellular iron is essential for the function of various enzymes, and excess iron is stored in ferritin to avoid oxidative damage; **4. Iron recycling in macrophages.** Iron from aged red blood cells is recycled by macrophages and exported via FPN. Unlike enterocytes, iron in macrophage plasma membrane is oxidized by ceruloplasmin before binding to transferrin; **5. Iron storage in the liver.** Excess iron is reversibly stored in the liver, and can be released into the circulation at conditions of low iron levels; **6. Iron regulation by hepcidin.** Systemic iron homeostasis is regulated by hepatocyte-secreted hepcidin. When iron becomes overload, hepcidin is up-regulated and induces FPN internalization and degradation in macrophages and enterocytes, thereby preventing excess iron into the bloodstream. Abbreviations: FPN, ferroportin; CP, ceruloplasmin; HEPH, Hephaestin; Hb, Hemoglobin; CYBRD1, cytochrome *b* reductase 1; DMT1, divalent cation transporter 1.

Iron in human body cycles among the serum, bone marrow, red blood cells, macrophages, liver, and various tissue cells, maintained at dynamic homeostasis. The proportion of iron deposited in these cells or organs differs greatly (Fig. 1). The body acquires iron from food supply by enterocytes in the intestinal lumen. Before absorption, exogenous iron is reduced to the ferrous form by cytochrome *b* reductase 1 (CYBRD1) (Fig. 2). Then, the ferric iron is translocated into enterocytes by divalent cation transporter 1 (DMT1), and exported into the blood by ferroportin (FPN). In mammalian cells, FPN is the sole iron exporter that is expressed in macrophages, enterocytes, hepatocytes, pancreatic cells, myocytes, bone marrow hematopoietic cells, and some neurons (Fig. 2). Before entering the blood, ferric ion is oxidized again into ferric form by Hephaestin [8]. In the blood serum, iron binds to transferrin and circulates throughout the body. A great proportion of total iron in the human body is bound to heme in erythrocytes to transport oxygen [9], and the remainder is mostly stored in the liver. Other tissue cells use a small amount of iron for their physical function. In addition to enterocytes, macrophages are another source of serum iron. When erythrocytes become senescent, they are phagocytosed by macrophages, and the erythrocyte iron is recycled and re-exported by FPN to fuel erythropoiesis in the bone marrow. In contrast to enterocytes, ferrous ion in macrophages is oxidized to ferric iron by ceruloplasmin before binding to transferrin [10].

Systemic iron homeostasis is regulated by hepcidin, the major iron-regulatory hormone secreted by hepatocytes (Fig. 2). Under iron overload conditions, hepcidin is upregulated and binds to FPN on enterocytes, macrophages, or hepatocytes, inducing FPN internalization and preventing the export of excess iron into the circulation [11]. Cellular iron is also maintained at dynamic homeostasis. Transferrin transports iron to various tissue cells, where it is recognized by the transferrin receptor 1 and internalized via endocytosis. Iron is then transported from endosomes to the cytosol via DMT1. Cytosolic iron is used by enzymes for their functions. Excess iron is oxidized and stored in ferritin [12] or exported to the serum via FPN.

## 3 Fenton reaction and the Nrf2 antioxidant system

In addition to constituting proteins or enzymes, there is also redox-active iron that cycles between the ferrous and ferric forms, acting as a redox regulator [13]. Ferrous iron can react directly with hydrogen peroxide (H_2_O_2_) to produce oxidants, a process called the Fenton reaction [14]. The products of Fenton reaction are highly reactive oxygen species (ROS), including hydroxyl free radicals (HO・) and hydroperoxyl free radicals (HO_2_・) [15]. These byproducts of Fenton reaction oxidize nucleic acids, proteins, and lipids, causing oxidative damage to cells. In addition to Fenton reaction, iron can indirectly induce the production of H_2_O_2_ and superoxide radicals (O_2_^・-^), by constituting many ROS-generating enzymes, including NADPH oxidases (NOXs), nitric oxide synthases (NOSs), and subunits of the mitochondrial electron transport chain [16]. Therefore, iron overload is associated with increased oxidative damage, leading to complications in rare iron overload diseases.

**Fig. 3:**
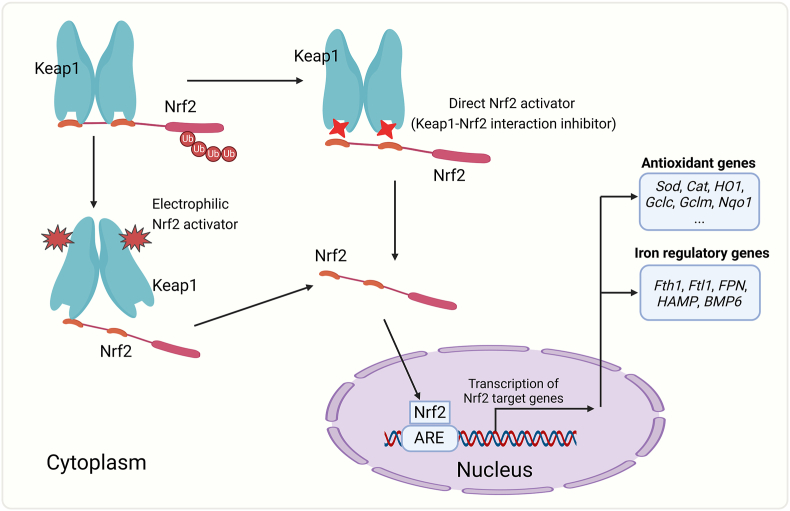
T**he Nrf2 antioxidant system and its activation by small activators.** Under normal conditions, Nrf2 binds to Keap1 in the cytoplasm and undergoes ubiquitination and degradation. Electrophilic Nrf2 activators can interact with the cysteine residues (primary C151) of Keap1 and induce conformation change of the protein, which disrupts its binding to Nrf2. Direct Nrf2 activator (Keap1 and Nrf2 interaction inhibitor) can direct occupy the Nrf2-binding domain in Keap1 and reduce Keap1-Nrf2 interaction. These two kinds of activators induce Nrf2 disassociation with Keap1 and promotes Nrf2 nuclear translocation. In the nucleus, Nrf2 binds to the ARE sequence of its target genes, including those involved in antioxidant responses or in iron homeostasis regulation. Abbreviations: ARE, antioxidant responsive elements.

To avoid damage from Fenton reaction or other oxidative stimuli, cells have developed a potent antioxidant system. Nrf2 is a core regulator of this antioxidant system. In a resting state, Nrf2 is sequestered by Keap1 in the cytoplasm and undergoes ubiquitination and proteasome degradation (Fig. 3). Under oxidative stress, Nrf2 is liberated from Keap1 and translocates into the nucleus, where it binds to the antioxidant responsive elements (ARE) in the promoter region of many antioxidant genes, including catalase, superoxide dismutase (SOD) and heme oxgenase-1 (HO1) [17]. SOD can reduce O_2_^・-^ to H_2_O_2_, and catalase is the mostly efficient enzyme to scavenge H_2_O_2_ without generating free radicals [18]. HO1 is an important antioxidant enzyme that degrades the pro-oxidant heme to prevent lipid peroxidation. Besides, Nrf2 can mobilize a more potent antioxidant system by activating many other enzymes, including Nqo1, Gclc and Gclm of the GSH metabolism system, which helps to scavenge ROS induced by iron overload.

Nrf2 can be activated by small molecules that affect the interaction between Keap1 and Nrf2 (Fig. 3). Many Nrf2 activators are electrophilic agents that can interact with the cysteine residues (primary C151) of Keap1 and induce conformation change of the protein, which decreases its binding to Nrf2 and reduces Nrf2 degradation. The clinically approved omaveloxolone and dimethyl fumarate are both electrophilic Nrf2 activators. In addition to Keap1, the electrophilic activity of these activators may also affect the function of other key enzymes or proteins, leading to off-target and side effects [19]. In human subjects, these activators are usually associated with elevated liver injury markers, including alanine aminotransferase, aspartate aminotransferase and γ-glutamyl transferase [20]. In contrast to electrophilic activators, small-molecule Nrf2 activators that directly disrupt KEAP1–NRF2 protein–protein interaction are under investigation [19]. However, there are currently no such direct Nrf2 activators being evaluated in clinical trials.

## 4 Regulation of iron metabolism by Nrf2

**Fig. 4:**
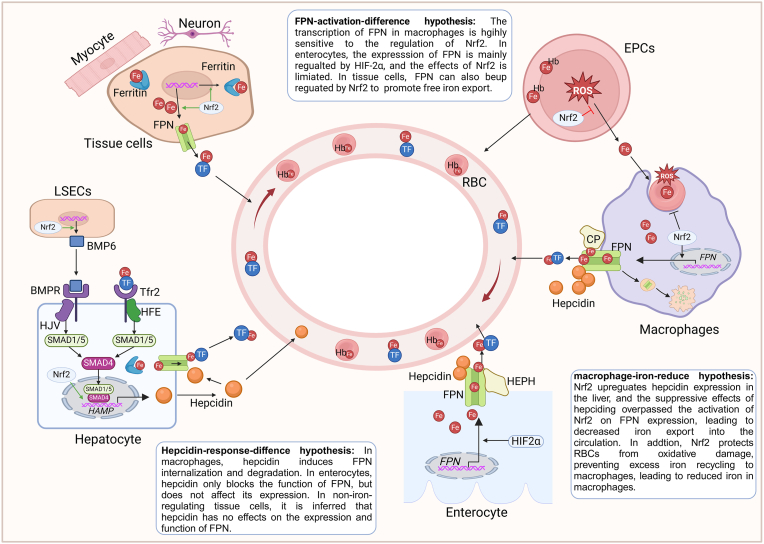
**Three hypotheses to account for the reducing effects on systemic and cellular iron levels in vivo.** First, upregulated hepcidin by Nrf2 activation reduces iron export from macrophages and enterocytes, but has no effect on iron export from various tissue cells. This is explained by the hepcidin-response-difference. Second, Nrf2 transactivates FPN expression to promote iron export from macrophages, enterocytes and various tissues. However, serum iron levels will not be increased due to increased FPN expression in these cells. This is explained by the “FPN-activation-difference” hypothesis. In combination to the “hepcidin-response-difference” hypothesis, it is inferred that Nrf2 activation decreases iron in various tissue cells, leads to a higher dynamic balance in macrophages, but has little effect on iron levels in enterocytes. Third, Nrf2 activation decreases iron levels in macrophages and this effect is explained by the “macrophage-iron-reduce” hypothesis. In conclusion, activation of Nrf2 will decrease iron levels in the plasma, macrophages and tissue cells. Abbreviations: FPN, ferroportin; CP, ceruloplasmin; HEPH, Hephaestin; LSECs, liver sinusoidal endothelial cells; Hb, Hemoglobin; EPCs, erythrocyte progenitor cells; HIF-2α, Hypoxia-inducible factor 2α.

In addition to antioxidant genes, Nrf2 also directly regulates iron homeostasis by affecting the expression of iron-related genes (Fig. 4). At the cellular level, Nrf2 transcriptionally activates the encoding gene of ferritin-L and ferritin-H [21,22], which form ferritin for iron storage. Besides, Nrf2 activates the transcription of *Slc40a1*, which encodes FPN, to promote iron export [23]. Both *Slc40a1* and ferritin genes contain the antioxidant responsive element (ARE) for Nrf2 binding [24]. By regulating these genes, Nrf2 reduces cytoplasmic iron and prevents oxidative damage caused by excess iron. At the systemic level, Nrf2 controls iron homeostasis by regulating the expression of Hepcidin Antimicrobial Peptide (*HAMP*) gene. *HAMP* encodes hepcidin. Its expression is regulated by a complicated signalling complex involving HFE/TFR2/HJV/BMP6/BMPR/Smads (Fig. 4). In the liver, liver sinusoidal endothelial cells secret bone morphogenetic protein 6 (BMP6), which binds to its receptors on hepatocytes and promotes *HAMP* expression in a paracrine manner [25]. Nrf2 enhances *HAMP* expression and upregulates hepcidin levels by directly binding to the ARE sequence in the promoter region of *HAMP* [26]. Besides, Nrf2 binds to a conserved ARE site in the BMP6 gene locus. The expression of BMP6, along with other Nrf2-regulated genes, is reduced in C2C12, MEF and L929 cells after Nrf2 silencing [27]. Through this ARE binding, Nrf2 induces BMP6 secretion from liver sinusoidal endothelial cells and enhances *HAMP* expression, thereby indirectly reducing systemic iron content [27].

Loss or activation of Nrf2 affects both systemic and cellular iron levels. Nrf2 knock-out (KO) mice fed an iron-rich diet accumulate significantly more iron in the liver [27]. In addition, Nrf2-KO mice have significantly higher serum iron levels than wildtype mice, accompanied by more iron accumulation in the liver and spleen, despite decreased iron absorption in the small intestine [28]. In addition to these iron-regulating organs, Nrf2-KO mice also accumulate iron in the hippocampus [29] and heart [30]. In contrast, pharmacological activation of Nrf2 in animal models reduces iron levels in the serum [27,29], hippocampus [29], liver [31,32], heart [33,34], brain [35], and bone tissue [36], indicating that activation of Nrf2 decreases both systemic and tissue cell iron content. This effect appears to be more pronounced under pathological conditions, particularly iron overload, as Nrf2-KO mice on a normal iron diet showed no changes on serum or liver iron content [27,37].

## 5 Hypotheses to account for the iron-decreasing effects of Nrf2

Nrf2 can activate FPN to promote iron export into the circulation, but it can also promote hepcidin expression to prevent FPN-mediated iron export. It remains unknown how Nrf2 coordinates these two contradictory processes to decrease systemic and cellular iron load. This phenomenon may be explained by three hypotheses (Fig. 4): 1) Differential sensitivity of FPN to hepcidin among iron-regulating cells. Macrophages are highly dynamic in iron releasing and recycling processes, and the response of FPN in macrophages to hepcidin is more sensitive than enterocytes [38]. FPN in macrophages exhibits a quick response (1–4 h) to Hepcidin, whereas in enterocytes the response is slow. Besides, Hepcidin can induce the internalization and degradation of FPN in macrophages, but it only blocks FPN function and has no effect on its protein level in enterocytes [39]. This difference may be attributed to the more developed phagocytosis and trafficking apparatus in macrophages. Accordingly, to circumvent expression and function redundancy, hepcidin may have no impact on FPN expression and function in various tissue cells, given that these cells contain only a minor proportion of total iron. Mobilizing iron in these cells will have negligible effects on systemic iron homeostasis [40]. This hypothesis is referred to as “**hepcidin-response-difference**”. **2)** Inconsistent activation of FPN by Nrf2 among cells. Nrf2 directly activates *FPN* transcription, but the extend of this regulation may vary among different cells. In macrophages, the expression of FPN is highly sensitive to Nrf2 activation. Our previous study indicated that *FPN* mRNA expression is the most significantly decreased among all the other genes in bone marrow macrophages from Nrf2 knockout mice, and Nrf2 activators can robustly increase *FPN* transcription [6]. In enterocytes, however, *FPN* expression appears to be more regulated by HIF-2α than Nrf2 [41]. Combined with the inhibition of hepcidin, Nrf2 is less likely to induce excess iron export from enterocytes to aggravate systemic iron overload. In other non-iron-regulating tissues, Nrf2 can also activate *FPN* expression [42]. with limited effects of hepcidin, FPN-mediated iron export dominates the effects after Nrf2 activation, thereby reducing intracellular iron levels [43]. This hypothesis is referred to as “**FPN-activation-difference**”; 3) Decreased iron recycling and export from macrophages. Pivotal for maintaining systemic iron homeostasis, macrophage FPN is highly sensitive to both Nrf2-mediated transactivation and hepcidin-mediated suppression. However, iron overload mice receiving Nrf2 activators showed decreased serum iron [27,29], suggesting that the overall effect of Nrf2 activation is to reduce macrophage iron export. This result indicates that hepcidin-mediated suppression overcomes Nrf2-mediated activation of FPN expression. Furthermore, iron overload promotes ROS production and oxidative damage in the body, which affects the physiology, morphology, and function of red blood cells (RBCs) [44], increases the proportion of abnormal RBCs [45], decreases RBC survival [44], and even triggers hemolysis [46]. This leads to increased iron recycling and iron content in macrophages. The loss of Nrf2 will exacerbate hemolysis in conditions of iron overload [28]. On the contrary, activation of Nrf2 can promote the expression of antioxidant genes in early precursor erythrocytes that possess a nucleus. Nrf2 activation also helps to reduce systemic oxidative stress, protects mature RBCs from systemic oxidative damage and reduces hemolysis [47,48], which in turn decreases excess iron recycling and prevents iron redistribution from RBCs to macrophages. Consequently, macrophage iron content will also be decreased following Nrf2 activation. This hypothesis is referred to as “**macrophage-iron-reduce**”.

The “hepcidin-response-difference” hypothesis helps to explain that Nrf2-promoted hepcidin expression suppresses iron export from macrophages and enterocytes, but does not affect iron export from various tissue cells. The “FPN-activation-difference” hypothesis explains that Nrf2 activation will not increase serum iron by promoting FPN-mediated iron export from enterocytes (due to alternative regulation by HIF-2α), but can promote iron export from various tissue cells. The “macrophage-iron-reduce” hypothesis is used to explain reduced iron redistribution from macrophages to other tissues and cells, as a result of decreased iron recycling from RBCs and iron export into the serum. Based on the three hypotheses, Nrf2 prevents cells from oxidative injury under iron overload conditions by two approaches, e.g. by reducing iron content and by enhancing antioxidant defense. The dual effects make Nrf2 activators very promising candidates for treating rare iron overload diseases. In the following section, we discussed the potentials of Nrf2 activators in treating these rare diseases, based on clinical and experimental evidences.

## 6 The use of Nrf2 activators in rare overload disorders

Iron overload is common in many rare diseases. We will talk about two categories of rare iron overload diseases. One category encompasses those caused by mutations of genes in iron metabolism, and the iron overload may be either systemic or tissue/cell-specific. Hereditary hemochromatosis is the typical type of systemic iron overload [49], while Friedreich ataxia exemplifies local iron overload, caused by iron misdistribution in the mitochondria [50]. The other category comprises rare diseases that have evidences of, or will be exacerbated by excess iron, but without direct causes of genetic abnormalities. For this category, we mainly focus on multiple sclerosis and EPP, because there are clinical evidences to favor the therapeutic effects of Nrf2 activators for their treatment. The potential therapeutic mechanisms of Nrf2 activators for these diseases are discussed based on experimental and clinical evidences.

### 6.1 Clinical evidences of Nrf2 activators for rare iron overload disease

#### 6.1.1 Multiple sclerosis

The first clinical evidence to support Nrf2 activators for rare iron overload diseases is the use of DMF in multiple sclerosis. Multiple sclerosis is a neurodegenerative disorder featuring damage to the myelin sheath and myelin-producing oligodendrocytes. Immune dysfunction, inflammatory responses, and oxidative injury are important causes of multiple sclerosis [51]. Over 80 % of patients experience alternating relapsing and remitting of multiple sclerosis [52]. DMF is approved for the treatment of relapsing forms of multiple sclerosis. The mechanisms underlying its action include modulation of immune responses and inhibition of oxidative stress [53]. Additionally, multiple sclerosis is characterized by dysregulated iron metabolism. Neuroimaging by susceptibility MRI showed increased iron deposition within deep grey matter structures [[54], [55], [56]], and the deposition is associated with disease progression [[55], [56], [57]]. Histopathological study showed abnormal iron deposition in microglia [58], the macrophages in the central nervous system. In physiological conditions, iron is primarily stored in oligodendrocytes and myelin sheaths. In multiple sclerosis lesions, iron is released from damaged oligodendrocytes and recycled by microglia, leading to iron accumulation in this cell population [59]. Microglia iron may also come from extravasated RBCs and infiltrating monocytes [60]. Increased iron amplifies microglia activation, leading to mitochondrial dysfunction, proinflammatory factor secretion and ROS production, which further exacerbates neuron damage and myelin breakdown.

As an Nrf2 activator, the action of DMF on multiple sclerosis may also be attributed to the reduction of intracellular iron in microglia. On the one hand, DMF may promote both iron export and iron storage in microglia via the Nrf2-*FPN* or Nrf2-*Ferritin* pathways. On the other hand, DMF reduces oxidative damage to RBCs and prevent excess iron recycling by microglia, according to the “macrophage-iron-reduce” hypothesis. Currently, studies exploring the effects of DMF on microglia iron metabolism are rare. A previous study revealed that DMF facilitates the uptake of Ferritin-H bound iron by microglia and maintains iron homeostasis in the brain [61]. This process is mediated by the upregulation of TIM-2, a surface receptor permitting Ferritin-H endocytosis [62], rather than by the canonical TFR1 receptor. TIM-2 mediated iron uptake is unlikely to induce intracellular iron overload, because iron is sequestered in Ferritin-H and kept in a less active status. In contrast, increased Ferritin-H uptake may contribute to the buffering of free iron and the reduction of ROS. Correspondingly, DMF-treated microglia exhibited an anti-inflammatory phenotype and a reduction in ROS production [61,63]. Notably, this iron-regulatory effect of DMF is Nrf2-independent. In conclusion, DMF may decrease free iron in microglia by promoting iron export and storage, preventing iron recycling from RBCs, and enhancing iron storage by increasing Ferritin-H uptake. This may, in turn, improve the symptoms of multiple sclerosis.

#### 6.1.2 Friedreich's ataxia (FA)

The efficacy of Nrf2 activators for rare iron-overload diseases is further supported by the approval of omaveloxolone for treating FA [64]. FA represents the most common hereditary ataxia of the European ancestry, accounting for approximately 50 % of all ataxia cases [65]. Patients present progressive ataxia, dysarthria, and multi-organ involvement. The average survival time following the onset of the disease is reduced to about 36 years, and cardiac complications are common causes of death [66,67]. The disease is caused by homozygous mutations of a GAA trinucleotide repeat expansion in the encoding gene of frataxin, an iron-binding protein that contributes to Fe–S cluster assembly in the mitochondria respiratory complexes [68]. Frataxin can also facilitate free iron efflux from the mitochondria into the cytosol [69]. Loss of functional Frataxin results in decreased iron efflux and increased iron retention in the organelle, leading to oxidative stress, inflammation responses and mitochondria dysfunction [70]. Frataxin is mostly expressed in spinal cord neurons, cardiomyocytes, and pancreas. These cells and organs are particularly vulnerable to mitochondrial damage, resulting in typical neuropathological symptoms, cardiac complications, and diabetes of FA.

Many therapeutic strategies have been proposed and investigated to treat FA, including antioxidants (idebenone, Coenzyme Q_10_, A0001, EPI-743, and Nrf2 activators), anti-inflammation (Methylprednisolone), iron chelation (Deferiprone), and gene therapies with the aim to increase Frataxin protein levels [50]. However, the majority of them are symptomatic treatments and none of them has succeeded in reversing or slowing the neurodegeneration of the disease. Antioxidant therapy is a main strategy for alleviating the symptoms of FA, but the therapeutic effects are controversial. Many direct antioxidant agents, including idebenone, Coenzyme Q10, A0001 (α-tocopherylquinone), fail to improve the neurological or cardiac outcomes of FA [71,72], suggesting that antioxidant strategy alone may not work effectively for the treatment of FA.

As the first FDA-approved drug for the treatment of FA, omaveloxolone has been shown to improve cardiac outcomes in animals of FA and neurological functions of patients with FA [64,73]. Omaveloxolone is a potent Nrf2 activator, which binds to the cysteines (primary C151) of the Keap1 protein and inactivates it, thereby liberating Nrf2 and activating Nrf2-related antioxidant proteins [74]. In addition to Nrf2 activation, omaveloxolone is also a NF-κB inhibitor [[75], [76], [77]] and can suppress inflammatory processes in mouse models of Alzheimer's disease [78]. In cell and mouse models of amyotrophic lateral sclerosis, omaveloxolone has been shown to inhibited ferroptosis by upregulating SLC7A11 and GPX4 expression [79]. These therapeutic effects make omaveloxolone very promising candidate for the treatment of neurodegenerative diseases.

FA is the only clinically approved indication of omaveloxolone, and the efficacy may not be solely attributed to the antioxidant effects after Nrf2 activation. On the contrary, the regulation on cellular iron homeostasis by Nrf2 may be more essential. The pathogenesis of FA is closely associated with mitochondrial iron overload. Nrf2 can facilitate iron storage or iron export by activating Ferritin H/L and FPN, respectively, which helps to reduce free iron in the neurons via the **“FPN-activation-difference”** effect. Therefore, the use of Nrf2 activators in FA will decrease the influx of cytosol iron into the mitochondria, thereby reducing excess mitochondria iron and preventing iron-induced toxic effects. This notion is supported by in vitro and in vivo experimental studies, which showed that Nrf2 induction can suppress iron-induced ferroptosis in the heart and ganglia of FA mouse models and in skin fibroblasts of human patients with FA [80,81]. The iron-decreasing effect, combined with the antioxidant capacity of Nrf2, confers dual beneficial effects on cells affected by Frataxin deficiency. The dual effects may help to explain why omaveloxolone is more effective than other direct antioxidants or iron chelators in the treatment of FA.

#### 6.1.3 Erythropoietic porphyria (EP)

**Fig. 5:**
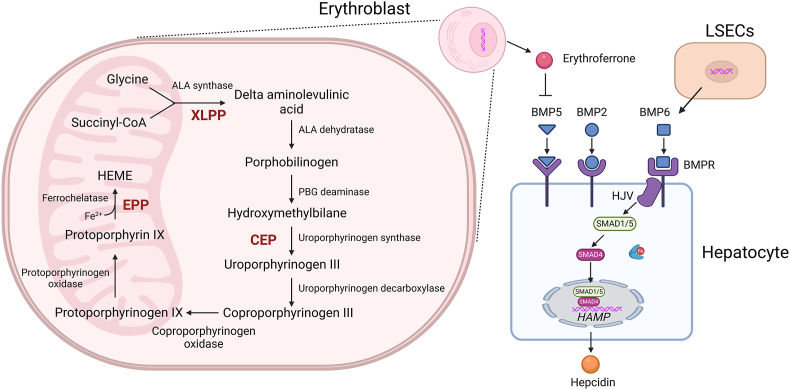
**Heme biogenesis, erythropoietic porphyria and ineffective hematopoiesis induced iron overload.** Heme biosynthesis starts with ALAS catalyzing the condensation of glycine and succinyl-CoA decarboxylation to form delta ALA. Gain-of-function mutations in ALAS2 cause XLPP. Two water molecules are then extracted from ALA by ALA dehydratase to form porphobilinogen (PBG). Uroporphyrinogen synthase (UROS) catalyzed the conversion of hydroxymethylbilin to uroporphyrinogen III after PBG deaminase catalyzes the conversion of PBG to hydroxymethylbilin. CEP is most often caused by a deficiency in UROS. The conversion of uroporphyrinogen Ⅲ to coproporphyrinogen Ⅲ is catalyzed by uroporphyrinogen decarboxylase. After catalyzing the conversion of coproporphyrinogen III to protoporphyrinogen IX, protoporphyrinogen oxidase catalyzed the conversion of protoporphyrinogen IX to protoporphyrin IX. Lastly, protoporphyrin IX and ferrous ion combine to form heme through the catalysis of ferrochelatase (FECH). EPP is characterized by FECH mutation that leads to reduced FECH catalytic activity. Ineffective hematopoiesis usually occurs when the heme biogenesis pathway is impaired. In this condition, erythroblasts secrete erythroferrone to inhibit the action of BMP5 on hepatocytes, thus inhibiting the secretion of hepcidin. Abbreviations: ALA, aminolavulinic acid; ALAS, ALA synthetase; XLPP, X-linked dominant protoporphyria (XLPP); CEP, congenital erythropoietic porphyria; EPP, erythropoietic protoporphyria; LSECs, liver sinusoidal endothelial cells.

The third clinical evidence comes from the efficacy of Bitopertin in the treatment of EP, a hereditary disease caused by mutations in genes involved in heme biosynthesis (Fig. 5). A key role of iron is to support heme and hemoglobin production. Disruption of heme biosynthesis in erythroid cells leads to porphyrin accumulation and deposition in the skin and many other tissues [82]. EP is divided into two distinct types depending on the mutated genes. The first type is congenital erythropoietic porphyria (CEP) caused by uroporphyrinogen III synthase (UROS) deficiency, leading to accumulation of uroporphyrins and coproporphyrins (Fig. 5). The second type is further divided into classical erythropoietic protoporphyria (EPP) caused by ferrochelatase (FECH) loss-of-function mutation, and X-linked dominant protoporphyria (XLPP) caused by aminolavulinic acid synthetase 2 (ALAS2) gain-of-function mutation [83]. These mutations result in protoporphyrin accumulation in erythroid cells. In addition to the mutated genes, the three types of EP also differ in their responses to iron status [84].

Induction of iron deficiency, including gastrointestinal bleeding [85] or iterative phlebotomies [86,87] has been shown to alleviate the symptoms of CEP. However, the responses of patients with EPP or XLPP to iron are completely different. Iron supplementation has been reported to benefit patients with XLPP, but worsen the symptoms in over 80 % of patients with EPP [84]. On the contrary, inducing iron deficiency by phlebotomy decreases protoporphyrin concentrations and improves the symptoms of patients with EPP [84]. These results can be explained by the regulation of intracellular iron levels on ALAS2 expression. Increased iron activates *ALAS2* transcription, promote the production of more intermediates, and increase protoporphyrin IX (PPIX) accumulation, whereas low iron levels inhibit it and suppress heme synthesis [84]. In the final step of heme biogenesis pathway, iron is incorporated into PPIX by ferrochelatase to produce Heme. In patients with *FECH* deficiency, the utilization of Fe^2+^ is reduced, resulting in a relative iron overload in the cells. The relatively increased iron activates *ALAS2* transcription, produces more intermediates and increases PPIX accumulation. Therefore, inducing iron deficiency is speculated to ameliorate iron overload and relieve the symptoms of EPP.

Based on this logic, reducing intracellular iron by promoting iron export may also confer therapeutic benefits to patients with EPP. As Nrf2 robustly transactivates FPN expression, Nrf2 activators may be potential candidates for the treatment of EPP. This assumption is supported by the clinical efficacy of bitopertin in treating EPP [4,88]. In mouse models of EPP, treatment with bitopertin reduced PPIX levels in RBCs [89]. In human subjects with EPP, bitopertin treatment reduced whole blood PPIX levels and improved photosensitive symptoms [4]. bitopertin is a glycine uptake inhibitor, but our recent study revealed that it is a potent Nrf2 activator and can reduce cellular iron by promoting *FPN* expression [6]. The antioxidant effects of bitopertin in erythroid cells further support its Nrf2 activating effect [47]. In vivo, oral bitopertin treatment increases hepcidin expression and diminishes liver iron overload in β-thalassemia mice [47], consistent with the in vivo effects of other Nrf2 activators on iron homeostasis [27,31,32]. Therefore, the efficacy of bitopertin in alleviating EPP symptoms may also be attributed to reduced iron in erythroid cells, via the **“FPN-activation-difference”** hypothesis of Nrf2 activation. Nrf2 activators may provide a new avenue for the treatment of EPP.

### 6.2 Potential use and precautions of Nrf2 activators for other rare iron overload diseases

In addition to the three successful diseases above, the potential use of Nrf2 activators for many other rare iron-overload diseases is discussed in the following section, based on the dual effects of Nrf2. The efficacy of Nrf2 activators, alone with iron depletion therapy, on these diseases are summarized in Table 1. It is notable that some iron disorders caused by FPN or Ceruloplasmin gene mutation may not be suitable indications of Nrf2 activators. For these diseases, precautions should be kept in mind, and the reasons are also discussed below.

**Table 1:** The efficacy of iron depletion therapies and Nrf2 activators on various rare iron overload diseases.

Rare iron overload diseases	Mutant genes	Type of iron overload	Efficacy of iron depletion therapy^a^	Potential efficacy of Nrf2 activators		References
				Efficacy	Possible mechanisms	
Multiple sclerosis		Focal iron deposition in the brain (microglia)	Ineffective with iron chelators	Effective; With clinical evidence	Promoting iron export by FPN and iron storage in Ferritin in microglia; Inhibiting excess iron recycling by microglia; Reducing oxidative damage.	[3]
Friedreich's Ataxia	*FXN*	Mitochondrial iron overload	Ineffective with iron chelators	Effective; With clinical and experimental evidence	Promoting iron export by FPN in tissue cells; Decreasing mitochondrial iron overload; Reducing oxidative damage	[2,80,81]
Erythropoietic porphyria	EPP: *FECH*	Relative iron overload in erythroid cells	Effective with iron chelators or phlebotomy	Effective; With clinical evidence	Promoting iron export by FPN in erythroid cells; Reducing oxidative damage	[[4], [5], [6]]
	CEP: *UROS*	Relative iron overload in erythroid cells	Effective with iron chelators or phlebotomy	May be effective; With clinical evidence		[[4], [5], [6]]
	XLPP: *ALAS2* gain of function mutation	No changes in iron levels	Ineffective; Effective with iron supplementation		The symptoms may be aggravated after iron decreasing by Nrf2 activators	
Neuroferritinopathy	*FTL*	Iron accumulation in cytosol	Ineffective with iron chelators or phlebotomy	Potentially effective;	Promoting iron export by activating FPN; Promoting iron storage by increasing Ferritin expression; Reducing oxidative damage	
Hemochromatosis	Type 1: *HFE*	Systemic iron overload	Effective with phlebotomy or iron chelators	Potentially effective; With animal evidence	Promoting iron export by FPN; Increasing Hepcidin expression; Inhibiting excess iron recycling by macrophages; Reducing oxidative damage	[27]
	Type 2: *HJV*, *HAMP*	Systemic iron overload	Effective with phlebotomy or iron chelators	Potentially effective;		
	Type 3: *TFR2*	Systemic iron overload	Effective with phlebotomy or iron chelators	Potentially effective;		
	Type 4: *FPN* gain-of-function mutation	Systemic iron overload	Effective with phlebotomy or iron chelators	Potentially ineffective; May even aggravate the iron overload status	Nrf2 activation promotes FPN expression and may even aggravate iron overload	
Congenital sideroblastic anemia^b^	*ALAS2*, *SLC25A38*, *STEAP3*, *ABCB7*, *HSPA9*, *PUS1*, *YARS2*	Mitochondrial iron overload^c^	Effective with phlebotomy	Potentially effective;	Promoting iron export by FPN in tissue cells; Increasing Hepcidin expression; Reducing oxidative damage	
Aceruloplasminemia	*CP*	Systemic iron overload	Ineffective in improving neurological symptoms with iron chelators or phlebotomy	May be inappropriate for treatment with Nrf2 activators	Iron cannot be exported from cells after Nrf2 activation due to deficient ferroxidase activity of CP	
Ferroportin disease	*FPN* Loss-of-function mutation	Iron overload in macrophages in the liver, spleen, and bone marrow	Effective with phlebotomy	May be inappropriate for treatment with Nrf2 activators	Iron cannot be exported from cells after Nrf2 activation due to the lack of iron-exportin activity in mutant FPN	[90]

Abbreviations: FPN, ferroportin; EPP, erythropoietic protoporphyria; XLPP, X-linked dominant protoporphyria; CEP, congenital erythropoietic porphyria.

a The efficacy is based on clinical evidences.

b Unlike XLPP, congenital sideroblastic anemia is caused by *ALS2* loss-of-function mutation.

c Patients may develop systemic iron overload due to erythroferrone overproduction and ineffective erythropoiesis.

#### 6.2.1 Neuroferritinopathy

Neuroferritinopathy is an inherited autosomal dominant neurological disorder caused by mutations in the L-ferritin (FTL) gene. The symptoms include focal dystonia, chorea, and movement disorders, which will progress to generalized dysphagia, aphonia, and motor disability in 5–10 years [91]. Neuroferritinopathy is an oxidative disorder of iron maldistribution in the brain [92]. Mutated L-ferritin is unable to incorporate iron for storage, leading to cellular accumulation of free iron and nonfunctional L-ferritin variants. This accumulation increases oxidative damage and induces ferroptosis [92,93]. Excess iron and L-ferritin variants are deposited in many tissues and organs, particularly in the central nervous system [94]. Therefore, neurodegenerative symptoms are characteristic of neuroferritinopathy. This is a very rare disease without effective treatments. Phlebotomy and iron chelators have been tried to treat neuroferritinopathy, but these iron depletion therapies fail to confer clinical benefits [91,95].

Similar to FA, Nrf2 activators may be effective for this disease. This assumption is based on three points regarding the pathology of neuroferritinopathy and Nrf2 functions: 1) free iron accumulation and consequent oxidative damage to brain tissue are typical pathological features of the disease; 2) Nrf2 have a potent antioxidant effect, thereby preventing the brain from oxidative damage; 3) according to the “FPN-activation-difference” hypothesis, Nrf2 may facilitate the export of cytosol iron from neurons, thereby reducing intracellular iron. Due to rare incidence of the disease, there is a lack of studies to support this assumption in cellular and animal models, as well as in human subjects, but the successful use of omaveloxolone for FA enhances our confidence in treating neuroferritinopathy with Nrf2 activators. In clinical practice, Nrf2 activators may be considered to compensate for the ineffectiveness of iron chelation therapies for neuroferritinopathy.

#### 6.2.2 Hereditary hemochromatosis (HH)

HH is a typical systemic iron overload disorder caused by genetic mutation-induced hepcidin deficiency. Mutations of any gene in the *HAMP* regulatory machinery result in decreased *HAMP* transcription (Fig. 4), which decreases hepcidin levels and causes iron overload. Depending on the mutated gene, HH is divided into four types [96]: type 1, the most common form caused by mutation of the homeostatic iron regulator (*HFE*) gene; type 2, subdivided into type 2A and 2B, caused by hemojuvelin BMP co-receptor (*HJV*) and *HAMP* gene mutations, respectively; type 3, caused by transferrin receptor 2 (*TFR2*) gene mutation; and type 4, caused by *FPN* gain-of-function mutation that leads to increased FPN activity. In patients with HH, excess serum iron accumulates in parenchymal cells, such as hepatocytes, cardiomyocytes and pancreatic cells, leading to oxidative damage. Currently, phlebotomy is used as the first-line therapy for the treatment of HH [97]. Erythrocytapheresis has also shown therapeutic effects for HH in several studies [98,99]. Iron chelators are second-line options, but the incidence of adverse effects is high [100]. These treatments help remove the excess iron from the body and improve hepatic, pancreatic and cardiac functions, but the organ complications (such as diabetes and arthropathy) are less likely to be reversed [101].

Nrf2 activators may also be potential therapeutic options for HH (Table 1). In mouse models of type 1 HH, treatment with the Nrf2 activator CDDO-Im decreased serum iron and reduced liver iron accumulation [27]. Mechanistically, Nrf2 facilitates Hepcidin expression by increasing BMP6 expression in liver sinusoidal endothelial cells (Fig. 4), which subsequently binds to its receptor (BMPR) and promotes *HAMP* transcription in hepatocytes [27]. Nrf2 can also bind to the promoter region of *HAMP* and directly activate *HAMP* transcription [26]. Furthermore, oxidative damage in HH mice is also decreased by CDDO-Im, indicating that both iron-decreasing and antioxidant roles contribute to the therapeutic efficacy of Nrf2 activators in HH. Currently, there is no evidence about the use of Nrf2 activators for type 2 and type 3 HH, but it can be speculated that Nrf2 activation will also have similar therapeutic benefits for the two types of HH, as they are also caused by hepcidin deficiency, despite their different gene mutations.

However, Nrf2 activators may not be suitable for type 4 HH, which is caused by increased FPN activity. In this condition, iron absorption by enterocytes and iron release into the circulation by macrophages are increased, leading to systemic iron overload. Increased FPN activity usually results from mutations that affect the hepcidin-binding site or ubiquitination site in FPN [90,102], which decreases hepcidin-induced FPN degradation. In this case, the "FPN-activation-difference" and "macrophage-iron-reduce" hypotheses of Nrf2 activation may not work in type 4 HH. Besides, as Nrf2 directly activates *FPN* expression [6], iron export into the bloodstream will be enhanced by Nrf2 activation, which may aggravate the iron overload conditions and increase disease severity. Although Nrf2 activation reduces iron overload-induced ROS generation, it remains unknown whether the benefits from this cytoprotective role can outweigh the risk associated with iron overload. Therefore, precautions are needed if Nrf2 activators are considered in type 4 HH.

#### 6.2.3 Congenital sideroblastic anemia (CSA)

CSA is an inherited disorder caused by mutations of genes involved in heme biosynthesis (ALAS2, SLC25A38, STEAP3), Fe–S cluster biosynthesis (ABCB7, HSPA9), or mitochondrial protein biosynthesis (mtDNA, PUS1, YARS2), leading to mitochondrial dysfunction and ineffective erythropoiesis in erythroid precursors [103]. ALAS2 mutation is the most common cause of CSA. Similar to Friedreich's ataxia, CSA is characterized by pathological iron accumulation in the mitochondria, but patients may progress to systemic iron overload due to overproduction of erythroferrone during ineffective erythropoiesis. Erythroferrone is an erythroblast-derived hepcidin-suppressing factor [104]. It decreases hepcidin levels by directing binding to BMP5 and interfering with the hepcidin-inducing effects of BMP5 [105]. Iron overload significantly increases the morbidity and mortality in patients with CSA by impairing hepatic and cardiac function. Vitamin B_6_ supplementation is a supportive therapy in patients with CSA, and the iron overload conditions can be managed by phlebotomy or iron chelators in patients with mild-to-moderate anemia [106].

Theoretically, activation of Nrf2 will benefit patients with CSA in terms of iron control (Table 1). Both BMP5 and BMP6 are strong regulators of hepcidin expression, and the hepcidin levels cannot be increased in BMP5 and BMP6 double-deficient mice as response to iron overload [105]. In CSA, the BMP5-hepcidin pathway is blocked by erythroferrone. However, this block can be overcome by activating BMP6-hepcidin axis, because BMP6 alone is able to ameliorate hemochromatosis in the absence of BMP5 [105]. As discussed in the above section, Nrf2 drives BMP6 expression in liver sinusoidal endothelial cells, and it also directly activates *HAMP* gene transcription to increase hepcidin expression, thereby decreasing systemic iron load. Combined with the iron export effects by FPN activation and antioxidant roles in erythroid precursors, Nrf2 activators are likely to protect patients from iron overload-induced cell and organ damage. It should be noted, however, that Nrf2 activators may not help to improve the anemia symptoms, but only help to prevent iron overload related secondary damages.

#### 6.2.4 Aceruloplasminemia

Aceruloplasminemia is a rare autosomal recessive, adult-onset disorder caused by mutations in the Ceruloplasmin (CP) gene. CP is a copper-containing protein with ferroxidase activity and antioxidant properties. Patients with this disease have both cerebral and systemic iron overload. Neurological symptoms are the dominant clinical features of aceruloplasminemia, while other organs are less affected, despite the iron overload status [107]. CP is expressed in two distinct isoforms: a plasma-soluble form secreted by hepatocytes, and a glycosylphosphatidylinositol (GPI)-anchored membrane form that is produced by various cells [108]. The GPI isoform of CP oxidates FPN-exported Fe^2+^ into Fe^3+^ on the plasma membrane to allow binding to extracellular transferrin. Mutations in the CP gene impairs the ferroxidase activity, leading to decreased iron export by FPN. Consequently, excess ferrous iron accumulates in tissue cells and induces oxidative damage, particularly in neurons. There are currently no curative treatments for aceruloplasminemia. Iron chelators can reduce systemic and cerebral iron overload, but cannot improve the neurological symptoms of aceruloplasminemia. Phlebotomy is even less effective than iron chelation. The inefficacy may be due to the fact that excess iron is trapped in tissue cells and cannot be exported in the absence of functional CP.

In this condition, Nrf2 activators are less likely to improve the iron overload status (Table 1). Hepcidin levels are increased in aceruloplasminemia [109], and activation of Nrf2 further increases hepcidin levels, which may even exacerbate iron trapping because hepcidin facilitates FPN degradation. Although Nrf2 activates FPN transcription, the iron export process cannot be enhanced due to the lack of CP. Therefore, precautions are needed when Nrf2 activators are considered for aceruloplasminemia. Nrf2 activation may compensate for the decreased antioxidant activity of CP mutants and may improve the neurological symptoms by reducing oxidative damage, but this assumption needs to be confirmed in clinical practice.

#### 6.2.5 Ferroportin disease

Ferroportin disease is caused by loss-of-function mutations in the *FPN* gene, leading to impaired iron export by cells. The typical pathological change is iron trapping in tissue macrophages in the liver, spleen, and bone marrow [90]. Iron overload in Kupffer cells is the hallmark of the disease, and iron deposition in hepatocytes is also present. Ferroportin disease is very rare, and the long-term outcome has yet to be evaluated. Over 80 % of patients with Ferroportin disease manifest hyperferritinemia, but only 11 % of them will progress to liver fibrosis or cirrhosis [110], suggesting that this type of liver iron overload is less severe than classical type 4 HH caused by FPN gain-of-function. Because iron is trapped in macrophages and cannot be released into the circulation and bone marrow, patients tend to develop overt anemia when the demands of erythropoiesis are increased (e.g. menarche) [90]. Phlebotomy is the cornerstone to treat patients with significant iron overload, but this approach is not well tolerated due to the risk of exacerbating anemia. The optimal goal of treatment is to reduce ferritin to the lowest acceptable level and to maintain normal hemoglobin levels. Due to the loss of FPN function in macrophages, activation of Nrf2 is less likely to extract the iron outside macrophages for redistribution. Therefore, the use of Nrf2 activators is not appropriate (Table 1). However, it can be carefully evaluated whether the antioxidant effects of Nrf2 activators are needed to improve patients’ symptoms in actual clinical settings.

## 7 An Nrf2 activator-based combination therapy for severe systemic iron overload

Iron chelators are the main non-invasive therapy to reduce systemic iron and play a fundamental role in the management of systemic iron overload. However, iron chelators are usually associated with serious adverse events [111]. In some conditions, monotherapy with iron chelators is not efficient to control severe systemic iron overload, and a combination of two or more iron chelators is used to maintain iron balance [112]. However, dual therapy with iron chelators greatly increases the risk of adverse effects and is not suitable for long-term use. As Nrf2 has both iron-decreasing and antioxidant effects, we propose a combination therapy with an Nrf2 activator and an iron chelator. This combination therapy may be more efficient than mono- or dual iron chelator therapy in many ways: 1) iron chelators can remove excess iron in various tissues and organs, and Nrf2 can protect RBCs from oxidative damage, thereby preventing iron redistribution from injured RBCs to macrophages and other tissues; 2) Nrf2 can both promote iron export from tissue cells and directly reduce ROS production, protecting cells from oxidative injury; 3) the hepcidin-activating and iron-decreasing effects of Nrf2 will help to decrease the dose of iron chelators and reduce the risk of side effects.

Animal studies have shown good efficacy of combination therapy with iron chelators and direct antioxidants for iron overload disorders [113,114]. Compared to direct antioxidants, Nrf2 activators have additional iron-decreasing effects and may be superior in iron overload conditions. This new combination therapy is suitable for systemic iron overload disorders, particularly HH. However, the efficacy of this combination therapy may be less effective in FPN gain- or loss-of-function related disorders, such as in type 4 HH, where the regulation of Nrf2 on the hepcidin-FPN axis is attenuated due to the insensitivity of mutated FPN to hepcidin; and in ferroportin diseases, where FPN has higher iron-exporting activity and may be even enhanced by Nrf2 activation in macrophages. This combination therapy is not suitable for disorders of iron misdistribution/local iron deposition, such as EPP, CSA, and FA, because patients with these disorders usually have normal systemic iron, and the conditions may even deteriorate after systemic iron chelation. For these diseases, monotherapy with Nrf2 activators may be an optimal choice, because Nrf2 may promote activate iron export in these tissues by activating FPN, according to the “FPN-activation-difference” hypothesis. This idea is supported by the clinical efficacy of DMF, omaveloxolone and bitopertin in treating multiple sclerosis, FA and EPP, respectively.

## 8 Conclusion

Nrf2 activators have both antioxidant and iron-decreasing effects, making them very promising candidates for the treatment of rare iron overload diseases. For in vivo regulation, we proposed three hypotheses to explain how Nrf2 reduces systemic and cellular iron levels. For clinical translation, we proposed a combination therapy with Nrf2 activator and iron chelator for selective systemic iron overload diseases. This combination therapy can decrease the dose of iron chelators, enhance the anoxidative effects and reduce the risk of adverse effects. Besides, monotherapy with Nrf2 activators may also be an ideal option for the treatment of iron misdistribution/local deposition disorders. Future studies may focus on evaluating the efficacy and promoting the clinical use of Nrf2 activators in more rare iron overload diseases.

## CRediT authorship contribution statement

**Yimin Dong:** Writing – review & editing, Writing – original draft. **Meng Zheng:** Writing – review & editing, Visualization. **Weizhong Ding:** Visualization. **Hanfeng Guan:** Writing – review & editing. **Jun Xiao:** Writing – review & editing, Supervision. **Feng Li:** Supervision, Project administration, Conceptualization.

## Declaration of competing interest

All the authors declared no conflicts of interest.

## Data Availability

No data was used for the research described in the article.
